# New York Letter

**Published:** 1893-07

**Authors:** W. Gwyer


### NEW YORK LETTER.

### A SURGICAL CLINIC,

HELD AT BELLEVUE HOSPITAL, NEW YORK CITY.

SERVICE OF DR. W. GWYER.

June —, 1893.

*Osteotomy.*—Patient was a girl two and a half years old. She had a congenital inward curve of the right leg, the curve being most marked at about the middle of the tibia. She had been in the hospital six months. The reason that she was not operated upon before was that her vitality had been too depressed to warrant the success of an operation. After a judicious course of treatment with cod-liver oil, iron, good food, exercise, fresh air and cleanliness, she was brought into a good state of general health.

The child's deformity was the result of the inner condyle of the femur having developed so rapidly, thus throwing the tibia outward, and as the child grew and used the limb the curve became more pronounced.

Fracturing a long bone for relief of deformity is beginning to be a frequent operation ; it is easy to perform, is attended with slight danger, and with good results. It can be done by making either a compound or simple fracture. By means of the osteo-closis, an instrument recently devised by Dr. Grattan, of Dublin, a simple fracture of the limb is made. This has many advantages and the apparatus is destined to meet with popular favor.

To determine where to break the limb, place the child on its back, with the legs close together, and notice the point where the deformity is most marked. This having been done with the child to be operated upon, it was decided to break the tibia at its center.

The limb was antiseptically prepared and a longitudinal incision, as long as the width of the chisel to be used, was made along the anterior border of the tibia. The chisel was introduced lengthwise, then turned when it was in the wound so as to cross the bone. With a wooden mallet it was driven three-fourths of the way through the bone. The chisel was removed and the limb broken. In reality, a compound green-stick fracture was made and the limb strengthened. The wound was then irrigated with bichloride, a couple of cat-gut sutures approximated the edges of the incision, and a plaster-of-Paris dressing applied, which will be left on for three weeks.

Such a case demands proper study and a careful adjustment of the fragments. Complete instrumental division of the bone is not made because of the danger of injury to the soft parts beneath, and because a green-stick fracture is desired. It is well to begin with a large chisel which should be laid aside for a smaller one when the bone is about half divided. Cases are reported where a spicular of bone has perforated the popliteal artery and caused death. This accident is avoided by making a green-stick fracture. Sepsis and injury to a large nerve or artery are the principal dangers. The prudent use of antiseptics does away with the former, and a skillful surgeon is hardly apt to injure a nerve or artery. As a rule, the results are good, the deformity is rectified, and no constitutional symptoms follow the operation.

*Thiersch Skin-grafting.*—Patient was a girl thirteen years old. Two months ago her hand was caught between the cog-wheels of a machine, and the skin, superficial vessels and nerves, and tissue down to the tendons on the back of the hand were destroyed. Nearly all of the phalanges and metatarsal bones of the hand were fractured. The hand was dressed antiseptically and put in a splint, and because of the efficient blood supply to the parts, the fractured bones healed, but a granulating surface about three inches square, was left on the dorsal surface of the hand.

The wound was scraped until the cicatricial layer of granulation tissue was reached. This layer is made up of flattened granulation cells, which are the first that form. Pressure of more recently formed granulation tissue above it flattens its cells and makes it of about the consistency of connective tissue. This layer must be exposed to obtain a successful result.

The antiseptic solutions that rendered the part aseptic, were washed away with a salt solution, a compress of hot sponges were held to the part for a minute to stop the bleeding.

The inner side of the thigh was then washed, shaved, and irrigated with bi-chloride, which was washed off with a salt solution. The skin was made tense with our hand, while a razor was applied and with a rapid to-and-fro movement a piece of the upper layer of skin was removed, nearly large enough to cover the wound on the back of the hand. This was left on the razor and brought to the hand where it was carefully placed over the denuded surface. Some small grafts were taken in the same way which filled in around the edges. Each graft was gently fixed in place and in contact with its neighbor by means of a small spatula. The hand was dressed by applying a compress moistened with salt solution and bandaged. The surface from which the grafts were removed was dusted with iodoform and dry cotton bandaged on.

This operation is becoming common. A pinkish color under the grafts at the end of a few days indicates that union is taking place, while if the grafts look white they will probably exfoliate. It has been proved in Bellevue Hospital that the best results are obtained by obtaining the required skin from the patient himself, and that the grafts taken from a member of his family unite better than if taken from a friend. Those sensational cases of several members of a masonic lodge, each sacrificing a portion of his integument to help to cover a brother's unhealed wound, are usually brilliant failures ; we hear of the operation much more frequently than we hear the result. The skin from a live frog, or the hairless skin of a puppy, or a rabbit, has been much used with partial success.

It is comparatively difficult to remove the grafts in the proper way. The method of doing it well can be acquired only by practice. When a part of the blade is felt to be running either too shallow or too deep, it is time to remove it. It doesn't make much difference if it goes a little deeper than it should, yet it is better not to remove any of the reticulated layer.

From the injury to the tendons, ligments and bones, all the joints of this patient's hands were immovable. By the continued use of massage, galvanism and passive motion, much movement can be obtained in these fingers, which, although far from perfect, will be of much more service to her than will be those of any artificial hand that can be applied.

